# Neural activity induced by sensory stimulation can drive large-scale cerebrospinal fluid flow during wakefulness in humans

**DOI:** 10.1371/journal.pbio.3002035

**Published:** 2023-03-30

**Authors:** Stephanie D. Williams, Beverly Setzer, Nina E. Fultz, Zenia Valdiviezo, Nicole Tacugue, Zachary Diamandis, Laura D. Lewis

**Affiliations:** 1 Department of Biomedical Engineering, Boston University, Boston, Massachusetts, United States of America; 2 Department of Psychological and Brain Sciences, Boston University, Boston, Massachusetts, United States of America; 3 Graduate Program in Neuroscience, Boston University, Boston, Massachusetts, United States of America; University of Rochester Medical Center, UNITED STATES

## Abstract

Cerebrospinal fluid (CSF) flow maintains healthy brain homeostasis, facilitating solute transport and the exchange of brain waste products. CSF flow is thus important for brain health, but the mechanisms that control its large-scale movement through the ventricles are not well understood. While it is well established that CSF flow is modulated by respiratory and cardiovascular dynamics, recent work has also demonstrated that neural activity is coupled to large waves of CSF flow in the ventricles during sleep. To test whether the temporal coupling between neural activity and CSF flow is in part due to a causal relationship, we investigated whether CSF flow could be induced by driving neural activity with intense visual stimulation. We manipulated neural activity with a flickering checkerboard visual stimulus and found that we could drive macroscopic CSF flow in the human brain. The timing and amplitude of CSF flow was matched to the visually evoked hemodynamic responses, suggesting neural activity can modulate CSF flow via neurovascular coupling. These results demonstrate that neural activity can contribute to driving CSF flow in the human brain and that the temporal dynamics of neurovascular coupling can explain this effect.

## Abstract 2

Cerebrospinal fluid flows around the brain to remove its waste products and maintain brain health. This study shows that driving neural activity via visual stimulation can rapidly induce large-scale fluid flow. Changes in brain hemodynamics can explain this pattern, providing a way to non-invasively manipulate brain fluid dynamics in humans.

## Introduction

Cerebrospinal fluid (CSF) circulates through the central nervous system, transporting solutes and clearing potentially harmful metabolic waste from brain tissue [1,2]. Reductions in CSF-mediated clearance of metabolites have been linked to aging, cognitive decline, and has been proposed as a biomarker of disorders such as Alzheimer’s disease [3–9]. Disrupted CSF flow has also been associated with a myriad of other neurological disorders [10]. Identifying the mechanisms that govern CSF flow is thus critical for understanding the role of these fluid dynamics in brain health and cognition.

Systemic physiological dynamics such as respiration and the cardiac cycle have long been known to drive CSF flow, as CSF pulses with each heartbeat and breath [11–16]. Recent work has also identified neuronal dynamics coupled to CSF flow, suggesting that neuronal activity could potentially implement an additional contributing mechanism to drive CSF flow, in concert with systemic drivers [17,18]. Specifically, large waves of macroscopic CSF flow appear in the ventricle during non-rapid eye movement sleep [17]. Slow waves of neural activity were followed by changes in cortical blood oxygenation and large-scale CSF inflow through the fourth ventricle. These coupled dynamics suggest that neural activity may act as an additional driver of macroscopic CSF flow due to neurovascular coupling: neurally induced changes in blood volume could displace CSF due to the fixed intracranial volume within the skull [19]. This mechanism would provide a way for the electrophysiological activity of neurons to directly control brain fluid flow via their effects on the vasculature. However, this hypothesized causal link between neural activity and fast, macroscopic CSF flow in the ventricles has not yet been established.

Previous work from both humans and animal models supports the existence of a neurally driven compensatory CSF flow mechanism. Local decreases in CSF volume have been observed during cerebral vasodilation evoked by various stimuli [20–22]. In addition, visual entrainment of arteriolar vasomotion during wakefulness can increase the clearance rate of solutes from the mouse brain [23]. However, whether neural activity induces large-scale CSF flow has not yet been tested. Furthermore, low-frequency neural activity during sleep is often coupled to autonomic state changes [24,25], which can lead to changes in vascular tone and CSF flow [26]. Whether neural activity itself can contribute to rapid changes in CSF flow via neurovascular coupling, separately from its associated low-frequency autonomic changes during sleep, has not yet been tested. We hypothesized that slow, large-amplitude changes in neural activity could drive CSF flow in the ventricles, by inducing neurovascular coupling.

Here, we manipulated neural activity in 3 independent experiments, each using high-intensity visual stimulation to test whether sensory-evoked neural activity could drive macroscopic CSF flow in the awake human brain. We used fast functional magnetic resonance imaging (fMRI) to simultaneously measure blood-oxygenation-level-dependent (BOLD) signals as well as CSF inflow in the fourth ventricle. Accelerated fMRI paradigms contain flow-related enhancement signals [27] in which high-velocity fluids produce bright signal in the outermost image slices. Our acquisition paradigm exploited these signals to measure upwards CSF flow, allowing us to image CSF movement while simultaneously acquiring hemodynamic BOLD data (S1 Fig). Importantly, this approach is only sensitive to upwards flow in the ventricle and does not measure the outwards (downward) fluid flow out of the fourth ventricle (S1 Fig).

First, in Experiment 1, we measured neural, hemodynamic, and CSF signals during visual stimulation using simultaneous electroencephalography (EEG)-fMRI at high temporal resolution. We established a clear temporal sequence of events during stimulation, in which neural activity recruited large-scale cortical BOLD changes, which alternated with macroscopic CSF flow. Next, in Experiments 2 and 3, we used independent fMRI datasets to replicate our findings from Experiment 1 and manipulated stimulus parameters to test whether this pattern was consistent with a mechanism based in neurovascular coupling. We found that stimulus duration*—*but not flicker frequency*—*was positively correlated with CSF flow, consistent with the temporal dynamics of neurovascular coupling serving as a key driver of CSF patterns. Finally, in Experiment 3, we found that macroscopic CSF flow is also coupled to systemic physiology, in parallel to the neurally driven flow during this high-intensity task condition. Together, our results show that large-scale changes in neural activity can drive macroscopic CSF flow in the human brain and elucidate the key properties of sensory stimulation that maximize flow.

## Results

### CSF inflow occurs at the offset of visual stimuli

**Fig 1:**
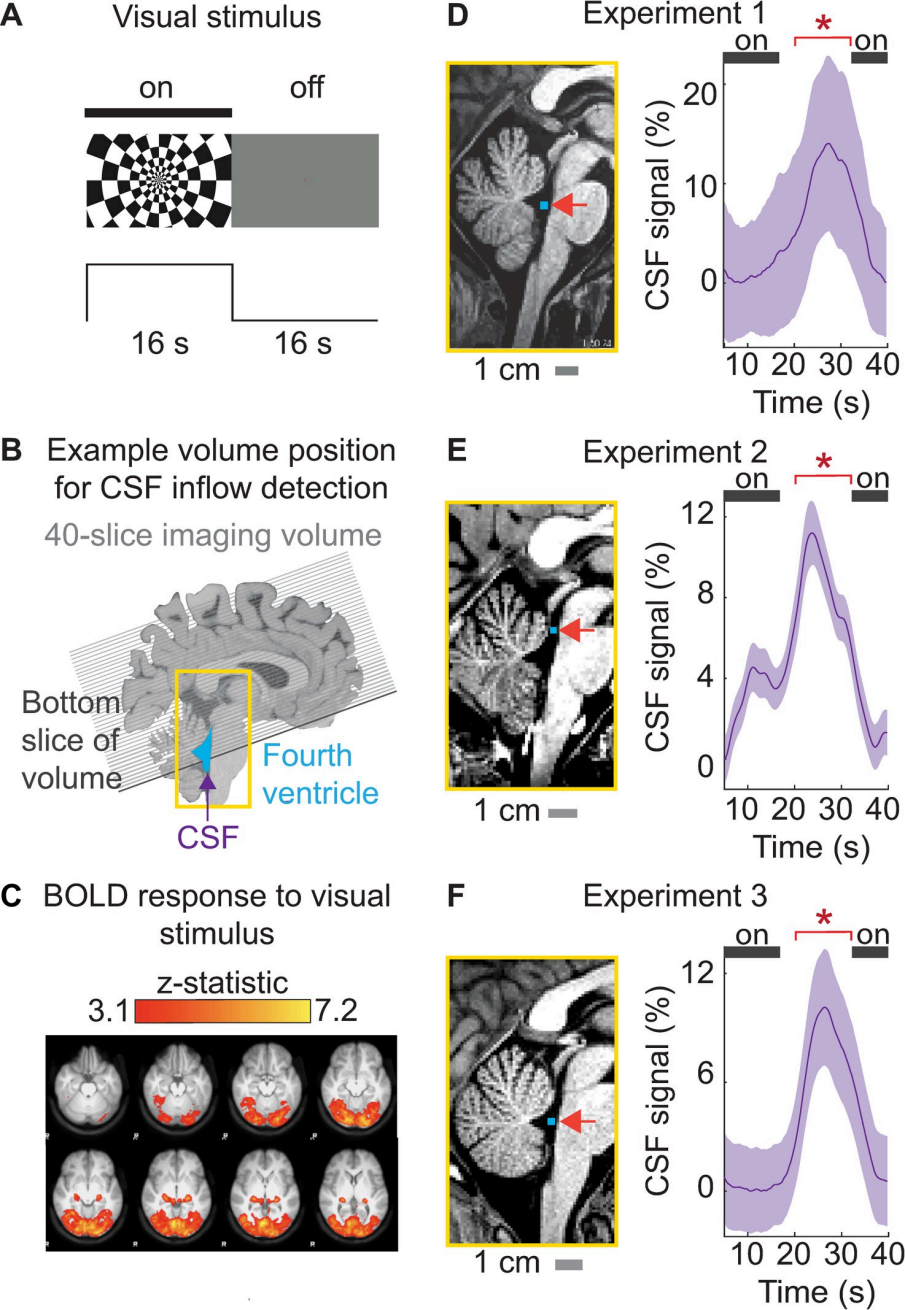
High-intensity visual stimulation drives significant cerebrospinal fluid inflow in the fourth ventricle and aqueduct. (**A**) Example frames from the block-design visual stimulus. “On” blocks consisted of a flickering radial checkerboard. “Off” blocks consisted of a gray screen. (**B**) The analysis extracted the CSF inflow signal measured in the fourth ventricle (blue) moving upwards (purple arrow) into the functional acquisition volume. The black line indicates an example position of the bottom slice of functional volume through the fourth ventricle, to enable upwards CSF flow detection. **(C)** Map in MNI space of widespread cortical activation in response to block-design checkerboard visual stimulus in *n* = 16 subjects in Experiment 3. Z-statistic values were thresholded at a level of 3.1 and corrected voxel-wise for multiple comparisons (*p* < 0.05). (**D**) **Left:** Example placement of CSF ROI (blue) in the fourth ventricle in 1 subject in Experiment 1. **Right:** Average evoked CSF inflow signal locked to the visual stimulus across subjects in Experiment 1 (*n* = 6). (**E**) **Left:** Example placement of the CSF ROI in Experiment 2, in the upper ventricle and aqueduct. **Right:** Average evoked CSF inflow signal across subjects in Experiment 2 (*n* = 20). (**F**) **Left:** Example placement of CSF ROI in Experiment 3, in the fourth ventricle. **Right:** Average evoked CSF inflow across subjects in Experiment 3 (*n* = 16). The black bar indicates “on” blocks. Shading indicates standard error across subjects. The time periods with significant increases in CSF flow signal amplitude are marked with * (*p* < 0.05, Wilcoxon signed-rank test). Traces are smoothed in a sliding 5-s window. Data from: doi:10.18112/openneuro.ds004478.v1.0.0 (Experiment 1), doi:10.18112/openneuro.ds004484.v1.0.0 (Experiment 2), and doi:10.18112/openneuro.ds004493.v1.0.0 (Experiment 3). BOLD, blood-oxygenation-level-dependent; CSF, cerebrospinal fluid; ROI, region of interest.

We manipulated neural activity to test whether sensory-evoked neural activity could induce CSF flow locked to a visual stimulus (Fig 1A). Prior work has shown that high-intensity visual stimulation induces substantial changes in cerebral blood volume [28,29]. We hypothesized that driving hemodynamic responses throughout a large expanse of cortex would also induce anticorrelated, compensatory task-locked CSF flow, as these changes in blood volume would need to alternate with changes in CSF volume to maintain constant intracranial pressure. We therefore selected a high-intensity visual stimulus that is known to reliably evoke a robust cortical hemodynamic response, a high-contrast flickering checkerboard presented in a slow block design [30–32]. We positioned the fMRI acquisition volume to simultaneously assess cortical BOLD and upwards CSF inflow through the fourth ventricle (Fig 1B). Next, we verified that the checkerboard visual stimulus engaged a large portion of cortex by identifying the voxels that showed a hemodynamic response to the visual stimuli, and found widespread hemodynamic responses to the stimulus, as expected (Fig 1C). The visual stimulus induced a significant response in 8.94% of voxels across the entire cortical gray matter, confirming that this high-intensity stimulus effectively drove large hemodynamic changes.

We then extracted CSF inflow signals from the fourth ventricle, allowing us to measure upwards CSF flow simultaneously with the cortical BOLD signal (S1 Fig). We investigated whether CSF inflow reliably appeared after stimulus offset, when the BOLD signal declines. In Experiment 1, we observed a significant evoked CSF response that peaked 11.5 s after stimulus offset (Fig 1D, mean change = 10.50%, *p* = 0.0312, Wilcoxon signed-rank test). This stimulus-locked flow signal was not driven by motion artifacts (S2 Fig). We then performed a replication analysis in 2 additional independent datasets using the same stimulus design. We first reanalyzed a previously published dataset using high spatial resolution 7T imaging to measure visual-evoked responses (Experiment 2), in which the acquisition paradigm allowed extraction of CSF flow in the upper ventricle and the aqueduct (Fig 1E). We investigated whether the stimulus-induced flow in fourth ventricle could be replicated in a different region, and again found stimulus-evoked CSF inflow with similar timing, locked to stimulus offset in Experiment 2 (Fig 1E, mean change = 6.00%, *p* < 0.0001, Wilcoxon signed-rank test). We conducted a third additional experiment and again replicated the visually induced CSF flow in the ventricle, in Experiment 3 (Fig 1F, mean change = 7.92%, *p* = 0.0004, Wilcoxon signed-rank test**)**. The results therefore replicated the finding that sensory stimuli induce large-scale CSF flow, and this property was robustly observed with distinct acquisition paradigms across 3 independent experiments.

To investigate the timing of this evoked flow across all experiments, we used a sliding window analysis and found a significant increase in upwards CSF flow between t = 18.5 s and 34.5 s, peaking at 25 s (i.e., 9 s after stimulus offset; S3 Fig). These results supported a mechanism of CSF flow in which it is driven by large changes in cortical hemodynamic signals. One key prediction of this mechanism is that stimulus trials with large cortical hemodynamic responses should induce higher CSF flow than trials with smaller cortical hemodynamic responses. We split trials in Experiment 3 into high- and low-flow trials and tested whether the magnitude of the cortical response co-varied with the magnitude of the evoked flow, defining high-flow trials as trials where flow exceeded the 95th percentile. The average cortical hemodynamic response for high-flow trials was indeed higher than the cortical hemodynamic response for low-flow trials (S4 Fig), confirming that hemodynamic and CSF flow magnitudes were correlated on a trial by trial basis. Together, these findings demonstrated that a visual stimulus designed to elicit widespread low-frequency neural activity and hemodynamic changes also causes large-scale CSF flow.

### CSF flow is coupled to neural and hemodynamic signals

**Fig 2:**
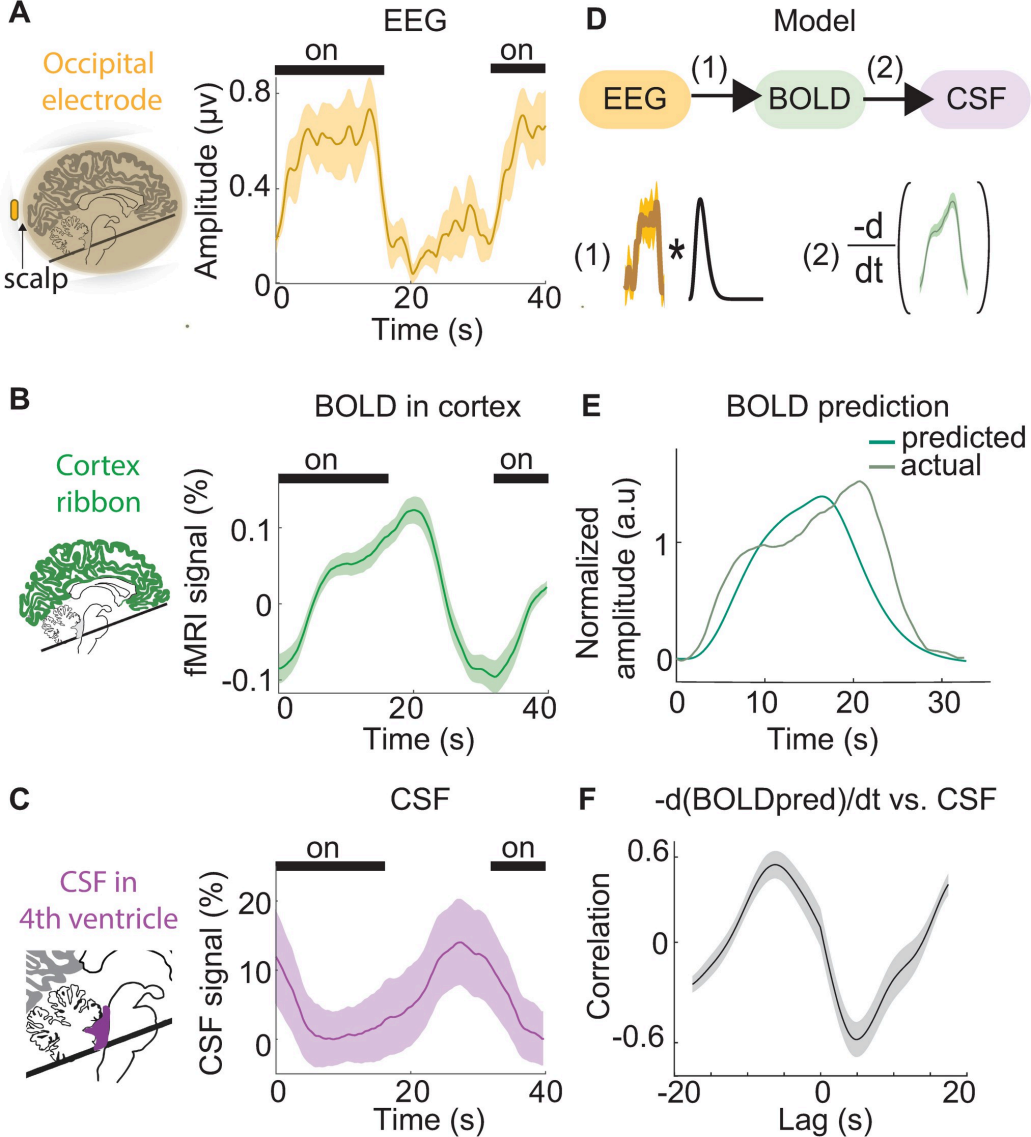
The temporal sequence of simultaneously measured neural, hemodynamic, and CSF signals. (**A**) The average amplitude envelope (orange) of stimulus-locked occipital EEG shows consistent neural responses during the visual stimulus (*n* = 12 runs, 6 subjects). (**B**) The average global cortical response (green) shows the stimulus induces a global increase in BOLD detectable in the cortical gray matter ROI. (**C)** The average CSF inflow signal (purple) shows consistent upwards flow after stimulus offset, when BOLD is decreasing. Shading in all panels indicates the standard error across 12 task runs. Black bars indicate stimulus ON blocks. Time t = 0 is the onset of a 16-s ON block. (**D**) A schematic of the model for predicting CSF flow using neural signals. The EEG envelope amplitude (orange) is convolved with a standard hemodynamic response function (black). The negative temporal derivative of the predicted hemodynamic signal (green) is then used to predict CSF flow. (**E**) The predicted BOLD signal produced by convolution overlaid on the actual BOLD signal. (**F**) The cross-correlation between the negative derivative of the predicted BOLD signal and CSF signal, showing that model predictions correlate with observed CSF flow. Shading is standard error across subjects (*n* = 6). Data from: doi:10.18112/openneuro.ds004478.v1.0.0. BOLD, blood-oxygenation-level-dependent; CSF, cerebrospinal fluid; EEG, electroencephalogram; ROI, region of interest.

To investigate the underlying mechanism of this visually evoked CSF flow, we next analyzed the simultaneous measurements of neural signals (using EEG), cortical hemodynamics (using BOLD), and CSF flow obtained in Experiment 1. A potential mechanism by which neural activity could drive CSF flow is through inducing changes in blood volume via neurovascular coupling, as large-scale changes in blood volume should be compensated for by CSF flow to maintain intracranial pressure [19]. Furthermore, high-intensity visual stimulation such as that used here is known to induce changes in cerebral blood volume [28,29]. Given that visual stimulation can induce an increase in cerebral blood volume, we hypothesized that intense visual stimulation could also induce rapid changes in CSF flow in the brain, driven by these vascular changes. We also predicted that a related measure of global hemodynamics, the global cortical BOLD signal, would alternate with CSF flow, consistent with previous evidence during sleep [17]. We therefore investigated the coupling between visually evoked neural activity, hemodynamics, and CSF flow. First, we verified that there was significant visually evoked neural activity in occipital EEG channels at the stimulus flicker frequency that occurred at stimulus offset (Figs 2A and S5; peak envelope amplitude = 0.69 μV). This neural response was followed by a subsequent BOLD response detected in the mean cortical gray matter signal (Fig 2B; unsmoothed peak amplitude = 0.28%), indicating that the visual drive was sufficient to induce a significant change in the global cortical signal. A pulse of CSF inflow through the fourth ventricle then occurred at stimulus offset (Fig 2C; peak unsmoothed amplitude = 14.7%).

To characterize the temporal relationships between the neural, hemodynamic, and CSF signals, we calculated the cross-correlation between the signals. We first evaluated the lag between the global cortical BOLD signal and the neural EEG signal and found that the global BOLD signal was maximally correlated with the EEG amplitude envelope at a lag of 6.5 s, which is consistent with the neurovascular coupling latency between neural and hemodynamic signals. The stimulus-locked CSF inflow signal was significantly anticorrelated with the task-evoked cortical BOLD signal (R = −0.62, *p* < 0.001, Pearson correlation). The CSF inflow signal was also anticorrelated with the amplitude envelope of the evoked occipital EEG signal (R = −0.66, *p* < 0.05, Pearson correlation). Examining this pattern across lags, we found that the CSF inflow signal was maximally anticorrelated (R = −0.85) with the BOLD signal at a lag of 3.25 s. To test our model of neurally driven CSF flow mediated by neurovascular coupling, we convolved the average visually evoked neural signal (i.e., the EEG envelope amplitude) with a standard hemodynamic response function to estimate the predicted BOLD signal, and then took the negative derivative of the estimated BOLD signal as the prediction of CSF flow (Fig 2D). We found that the experimental CSF signal aligned with the CSF model prediction (maximal R = 0.46, Pearson correlation). These results thus demonstrated a sequence of events consistent with the hypothesis of neurally driven CSF flow, mediated by global hemodynamic changes. First, occipital EEG power at the stimulus frequency increased at stimulus onset, signaling widespread visual-evoked neural activity. Next, the global cortical BOLD signal increased, while CSF inflow was suppressed. Lastly, at stimulus offset, the BOLD signal declined and CSF flowed upwards into the fourth ventricle.

### The evoked CSF signal matches global cortical BOLD responses across a wide range of stimulus parameters

Our results from Experiment 1 confirmed that the timing of CSF flow was consistent with a neural mechanism mediated by hemodynamics. This hypothesis of neurovascular-coupling-driven CSF flow led to a subsequent prediction: Stimuli that induce large hemodynamic differences should have large effects on CSF flow, whereas modulating neural activity without altering hemodynamics should have minimal effects on CSF flow. To investigate this possibility, we next tested whether altering the stimulus parameters could modulate the timing and magnitude of CSF flow, in Experiments 2 and 3.

**Fig 3:**
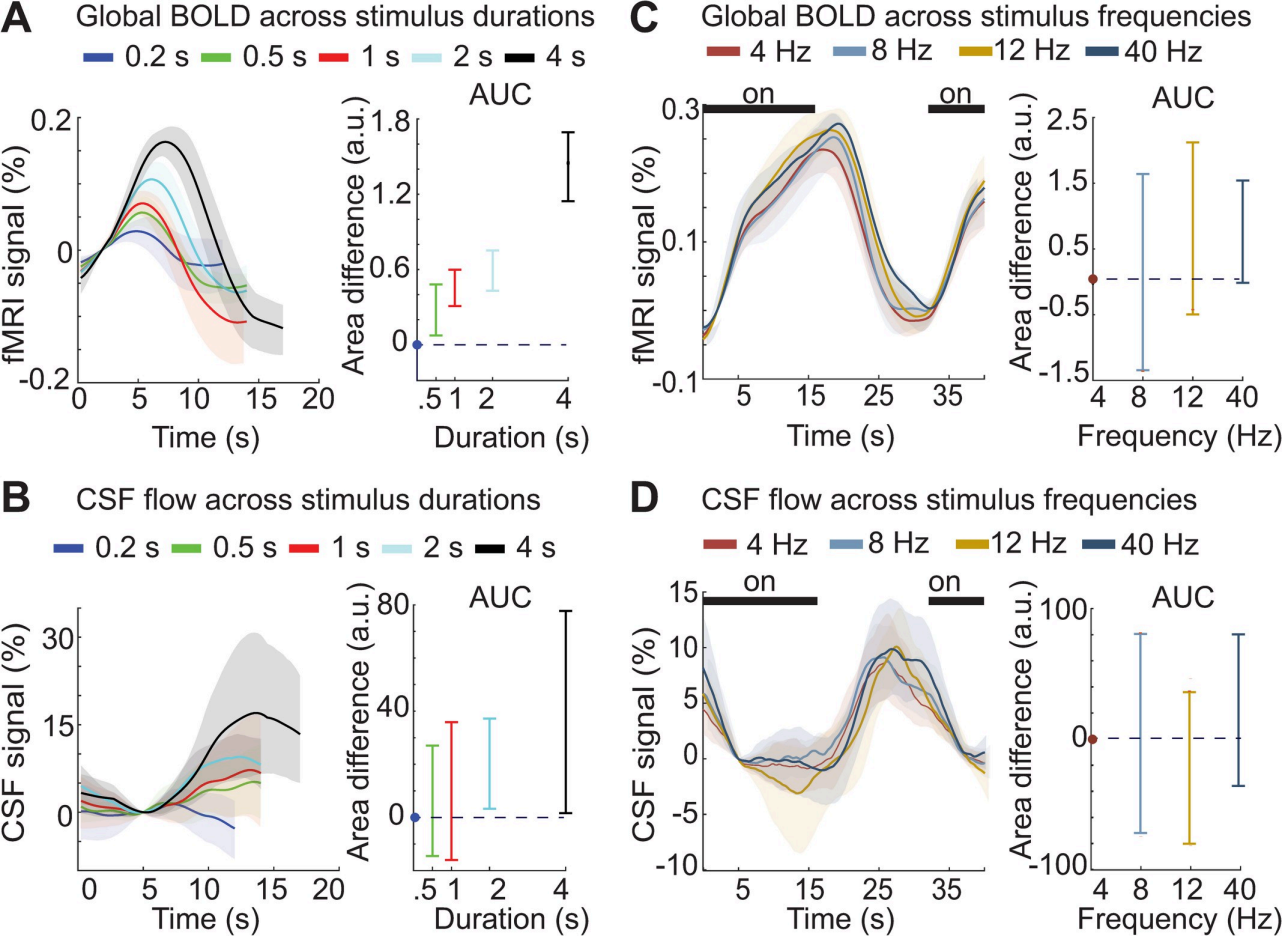
CSF responses match cortical hemodynamic responses across a range of stimulus parameters. (**A**) **Left**: Traces show the mean deconvolved cortical responses across subjects for each stimulus duration (*n* = 105 runs, 12 subjects). Colors represent the duration (0.17–4 s) of ON blocks, consisting of a 12-Hz flickering checkboard. Shading represents the 95% confidence intervals (bootstrap analysis). **Right**: The average area under the curve of the cortical BOLD response increases as a function of stimulus duration. Error bars represent the 95% confidence interval (bootstrap analysis, Bonferroni corrected). (**B**) **Left:** Traces are the mean deconvolved CSF flow signals for each stimulus duration. Shading represents the 95% confidence intervals (bootstrap analysis). **Right**: The average area under the curve of the CSF response increases as a function of stimulus duration, matching the pattern seen in BOLD. Error bars represent the 95% confidence interval (bootstrap analysis, Bonferroni corrected). (**C**) **Left:** Average stimulus-locked cortical responses across stimulus flicker frequencies. Shading indicates standard error across subjects (*n* = 143 runs, 16 subjects). **Right**: Average difference in the area under the curve of the cortical BOLD response for the 3 higher stimulus frequencies with the 4-Hz condition. Error bars show the 95% confidence intervals (bootstrap, Bonferroni corrected). (**D) Left:** Average stimulus-locked CSF responses also show no significant change across flicker frequencies. Shading indicates standard error across subjects. **Right**: Average area under the curve for the CSF responses does not change significantly across stimulus frequencies. Error bars represent the 95% confidence interval (bootstrap, Bonferroni corrected). Dashed lines indicate y = 0; dots indicate the value used to normalize area for each subject, which was the shortest duration (0.17 s) or the lowest frequency (4 Hz). Data from: doi:10.18112/openneuro.ds004484.v1.0.0 (Experiment 2), doi:10.18112/openneuro.ds004493.v1.0.0 (Experiment 3). BOLD, blood-oxygenation-level-dependent; CSF, cerebrospinal fluid.

Changing the duration of visual stimuli has profound effects on hemodynamic responses, with short (<1 s) stimuli inducing far smaller BOLD responses than long (16 s) stimuli [33–37]. We therefore investigated the CSF responses to stimuli of varying duration in Experiment 2 (durations = 0.17, 0.5, 1, 2, 4, and 16 s). We first examined the evoked BOLD response throughout the entire cortical gray matter, as this global measure is most relevant to assess the brain-wide hemodynamic changes hypothesized to drive large-scale CSF flow in the ventricle. As expected, we observed a strong relationship between the duration of the ON block and the magnitude of the global cortical BOLD signal (Fig 3A), with longer duration stimuli evoking significantly larger global cortical BOLD responses (Fig 3A; 95% CI for the area difference between each stimulus duration and the shortest duration: [0.060, 0.49], [0.30, 0.62], [0.43, 0.73], [1.14, 1.65], Bonferroni corrected). We tested whether the evoked BOLD signals were significantly different from baseline signals and found that all stimulus durations evoked significant cortical BOLD responses (*p* < 0.01 paired *t* test). The evoked CSF flow was also significantly higher than baseline flow for all stimulus durations (*p* < 0.01, paired *t* test) except for the 0.17 s duration, which was not significant (*p* = 0.45). We next tested how CSF flow changed across stimulus durations and observed significantly larger CSF inflow responses to longer stimuli (Fig 3B; 95% CI for the area difference between each stimulus duration and the shortest duration: [−15.07, 25.49], [−17.06, 35.12], [0.96, 38.26], [1.04, 78.26], Bonferroni corrected). The relationship between stimulus duration and amplitude of the evoked response was strongly similar in both the BOLD and CSF signals, with larger hemodynamic responses coupled to higher CSF flow. Sensory stimulation that induced larger global BOLD responses thus also induced greater CSF flow in the fourth ventricle.

We next investigated whether changing the flicker frequency of the stimulus would modulate CSF flow, as this substantially alters the timing of neural activity but with relatively small effects on hemodynamic responses. The 8 to 12 Hz frequency range typically evokes the greatest hemodynamic responses in visual regions, in both fNIRS and fMRI studies [31,32]. Consistent with this prior work, in primary visual cortex, we consistently saw larger cortical BOLD changes evoked by an 8-Hz, 12-Hz, or 40-Hz stimulus than for a 4-Hz stimulus (95% CI for the difference in AUC of V1 between each frequency and 4 Hz: [0.69, 6.62], [2.95, 9.37], [2.86, 8.68] for 8, 12, 40 Hz, respectively; Bonferroni corrected). In contrast, although a slight trend may have been apparent, we did not find a significant difference between stimulus flicker frequencies for the global cortical BOLD signal, suggesting that brain-wide hemodynamic effects were similar despite focal differences in visual cortex (Fig 3C, 95% CI for the difference in the AUC of cortex between each frequency and 4 Hz: [−1.35, 1.65], [−0.51, 2.12], [−0.02, 1.55], Bonferroni corrected**)**. We found an analogous pattern in the ventricle: the CSF signal amplitude also did not differ across stimulus frequencies (Fig 3D, 95% CI for AUC of CSF: [−72.86, 81.24], [−81.09, 36.61], [−36.80, 80.99], Bonferroni corrected). The visual stimuli used in this experiment thus did not induce detectable frequency-dependent hemodynamic differences at the global cortical scale (despite local differences in visual cortex), and furthermore produced similar evoked CSF responses. We therefore concluded that manipulating neural activity in ways that preserved overall global hemodynamics in turn preserved stimulus-evoked CSF flow.

### CSF flow driven by systemic physiology is distinct from neurally driven CSF flow

**Fig 4:**
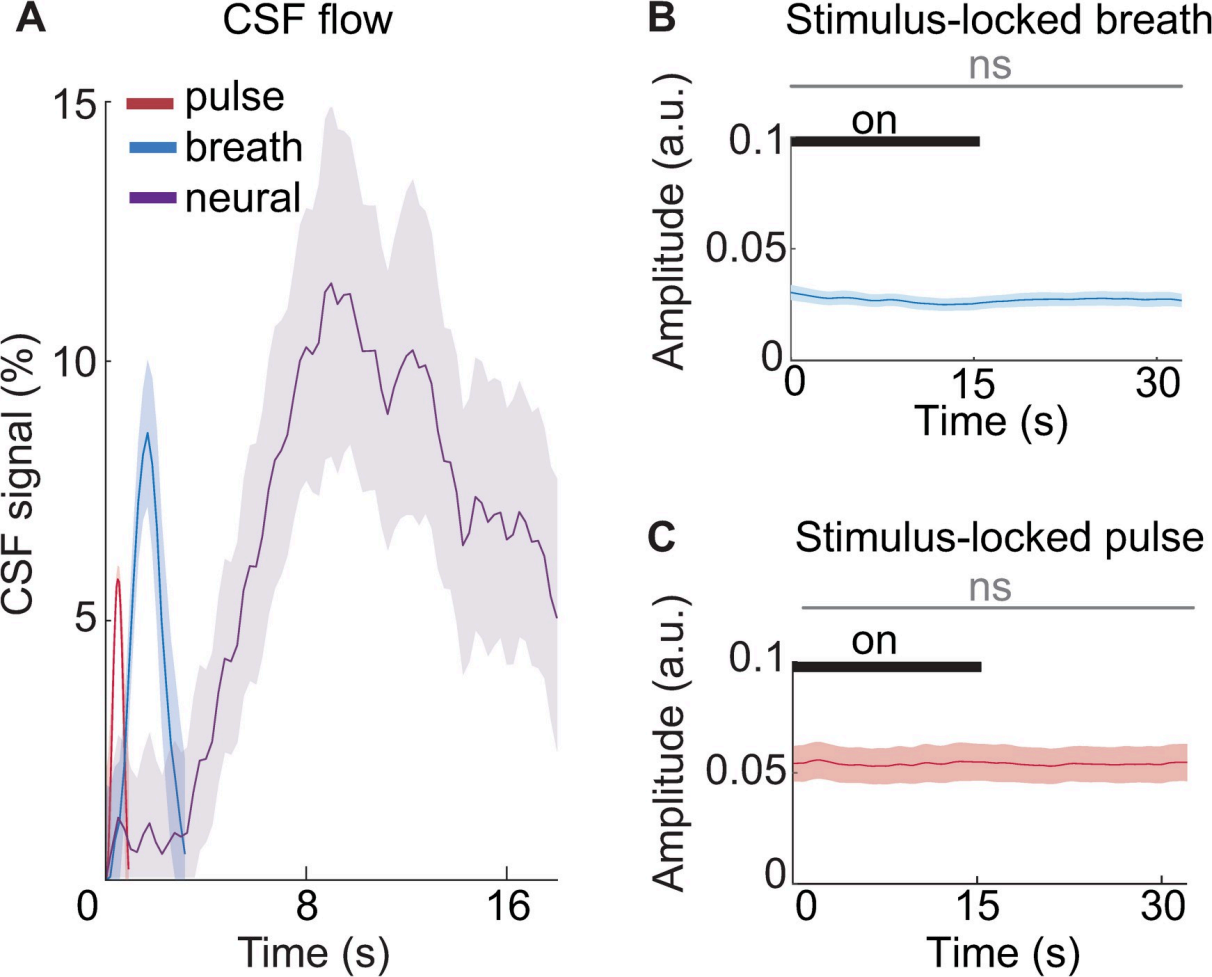
Neurally driven CSF flow signals during the visual task have comparable magnitude to flow signals driven by systemic physiology. (**A**) Average CSF flow locked to breath cycle (blue), cardiac cycle (red), and visual stimulus offset (purple), with no temporal smoothing. The breath and cardiac cycles were scaled by the average length of a single cycle (average breath cycle length = 3.16 s; average cardiac cycle length = 0.9 s). Shading is standard error across subjects (*n* = 16). (**B)** The average amplitude envelope of the respiration signal shows no significant change linked to the visual stimulus. Shading indicates standard error across subjects (*n* = 16). (**C**) The average amplitude envelope of the pulse oximeter signal shows no significant change locked to the stimulus. Shading indicates standard error across subjects (*n* = 16). The gray bar indicates that variations in the signal amplitude between stimulus on and off blocks were not statistically significant. Data from: doi:10.18112/openneuro.ds004493.v1.0.0. CSF, cerebrospinal fluid.

We next investigated whether systemic physiology, rather than neurovascular coupling, could have induced these sensory-evoked CSF effects. Systemic physiological factors, such as systemic vasoconstriction and cardiovascular pulsatility, can drive widespread BOLD changes [38–42], leading us to test whether systemic physiology covaried with our manipulations of neural activity. To test whether there were cardiac or breath-locked dynamics that could explain the high-amplitude pulses of CSF flow that we observed, we acquired pulse oximetry and respiratory data during Experiment 3. We extracted the average CSF inflow signal locked to the breath and cardiac cycles during the visual task and compared it with the CSF flow locked to neural activity. We found that CSF signals were indeed strongly locked to systemic physiological rhythms, with smaller amplitude signals (cardiac-locked signal = 5.8%; breath-locked signal = 8.6%) than the CSF signal driven by neural activity in this task (stimulus-locked signal = 11.5%; Fig 4A, *p* < 0.001, ANOVA). In addition, since autonomic changes contribute to CSF flow [26], we also examined whether there were any significant stimulus-driven changes in autonomic state detected in the amplitude envelope of the respiratory and cardiac signals (Fig 4B and 4C). We found no significant difference in the amplitude of the breath signal across stimulus on and off blocks (mean difference = −0.0004, 95% CI [−0.002, 0.0007]), nor in the amplitude of the pulse oximetry signal (mean difference = 0.0001, 95% CI [−0.001, 0.001]). This finding further supported our hypothesis that a neural mechanism, in addition to the previously identified systemic mechanisms, induces macroscopic CSF flow during sensory stimulation.

## Discussion

We tested whether a neural mechanism contributes to CSF flow in the awake human brain, using fast imaging to investigate the temporal dynamics of neural, hemodynamic, and CSF signals. Across three experiments, we found that neural activity evoked by high-intensity visual stimulation drove macroscopic CSF flow in the fourth ventricle. We identified a sequence of events that led to CSF flow, in which neural activity recruited widespread cortical BOLD changes, which were followed by CSF inflow. Furthermore, we found that modulating visual stimulus parameters consistently caused CSF flow responses that matched hemodynamic responses. These findings supported our hypothesis that neurovascular coupling that induces sufficiently large changes in blood volume—and in turn changes in the global BOLD signal—can drive waves of CSF flow.

Our results from the simultaneous EEG-fMRI measurements in Experiment 1 suggested that CSF flow could be driven by manipulations of neural activity with high-intensity visual stimuli. In Experiments 2 and 3, we modulated the hemodynamic response dynamics by modifying visual stimulus parameters. Altering the duration of the stimulus profoundly affected CSF responses, with patterns consistently mirroring the hemodynamic response for each stimulus condition. However, altering the stimulus frequency of the visual stimulus did not significantly alter the global cortical signal response (despite inducing distinct responses in visual cortex), nor the magnitude of CSF inflow. This finding is consistent with our hypothesis that CSF flow is tightly coupled to cerebral blood volume changes driven by neural activity, when changes in neural activity are sufficiently large and coherent across the brain.

In keeping with this idea, our results are consistent with neural activity being one of multiple drivers of CSF flow, as under this model, any large-scale modulator of cerebral blood volume will also be expected to induce CSF flow. The effects of the cardiac and respiratory rhythms on CSF flow are well established [13–15]. Furthermore, low-frequency vascular oscillations have been found to be coupled to CSF flow during wakefulness [43]. The relative contributions of each mechanism likely depend on brain state: for example, neurovascular drivers may dominate when widespread high-amplitude, low-frequency (<0.2 Hz) neural activity is present (whether spontaneous or sensory driven), while autonomic and systemic contributors may play a larger role in states with autonomic arousals such as light sleep [26], or during a paced breathing task [44]. Importantly, while neurovascular coupling was a major driver of CSF flow during the intense visual stimuli used here, its relative contribution may be substantially lower during more naturalistic stimulus paradigms or in the resting state.

Disentangling these mechanisms in resting-state, spontaneous data can be challenging due to the frequent collinearity of neural activity with systemic physiological state changes, which precludes simple regression models. Our use of sensory-evoked neural activity allowed us to separately investigate the contributions of physiological drivers of flow and neural drivers of flow, establishing a proof-of-concept. Our work thus highlights that neurovascular coupling is indeed one of the causal contributors to macroscopic CSF flow movement and can exert large effects on CSF flow even during wakefulness if a high-intensity, slow task design is used.

Future work could investigate whether alternate stimuli, such as multisensory stimuli that engage larger swaths of cortex, could be used to manipulate CSF flow. Research in mice has shown that 40-Hz gamma entrainment with audiovisual stimulation can reduce phosphorylated tau and amyloid in mice [45–47]. Gamma entrainment is expected to drive robust hemodynamic responses when its envelope amplitude is modulated at ultra-slow rates (e.g., 0.1 Hz) [48]. Our results therefore suggest that one potential mechanism for stimulus-induced reduction of pathology may be the induction of CSF flow; however, we found that 40-Hz stimulation was not unique in this respect, as it evoked CSF flow that was similar in magnitude to other frequencies in this stimulus paradigm. It is possible that multisensory gamma stimulation, instead of the visual-only stimulation used here, may have a more robust effect on the global cortical signal, and may drive greater CSF flow. Similarly, it is possible that stimulation delivered at even slower block rates could drive greater CSF flow.

A key next question is what the physiological consequences of this macroscopic CSF flow are for waste clearance, as these experiments did not include measures of endogenous metabolite clearance, and the solute transport mechanisms of the brain remain a topic of debate [23,49–52]. Computational fluid dynamic simulations have shown that functional hyperemia could contribute to metabolite clearance by increasing the mixing of CSF in the perivascular and subarachnoid compartments [53]. However, the functional consequence of macroscopic CSF flow in the ventricles is not yet empirically established and was not measured in this study. Direct measurements of solute clearance in humans will ultimately be necessary to clarify how large-scale macroscopic flow influences solute clearance. Intrathecal injections of contrast agents have recently been successfully used in humans to quantify CSF tracer transport [54], and future research could therefore test whether neurally driven CSF flow alters clearance in humans.

Our results raise the intriguing question of why neural activity would be coupled to fluid flow—is this coupling an epiphenomenon, or does it serve a functional purpose to link these electrical and fluid systems? One possibility is that this mechanism enables neurons to exert spatial control over solute transport and fluid flow, with coordinated local neuronal activity inducing higher flow rates. In this scenario, neurons that were most active could theoretically induce higher local solute clearance when they cease firing. However, CSF flow was most tightly predicted by global hemodynamics, which are not always directly coupled to neuronal metabolic rate, as many large changes in neuronal activity will not necessarily induce a large hemodynamic response. In this scenario, the systemic drivers of CSF flow could act as a parallel mechanism to ensure fluid flow in cases where neurovascular coupling-driven flow is altered or reduced, such as in natural aging or in disease. Future work will be needed to investigate the functional consequences of this coupling.

Taken together, these results demonstrate that CSF flow can be driven by inducing high-intensity visually evoked neural activity during the awake state in humans. We conclude that in addition to the well-established effects of systemic physiological factors such as respiration and vasoconstriction on CSF flow, neural activity and neurovascular coupling is an additional contributing mechanism that can drive fast, large-scale changes in CSF flow. Furthermore, our noninvasive approach provides an avenue to now test the integrity of this mechanism in clinical populations, to ultimately understand its consequences for brain function.

## Methods

### Data acquisition and stimulus design

#### Data acquisition—Experiment 1

Six healthy adults (4 female; age range 24 to 33 years, mean = 27.16) were included. All subjects gave written informed consent to procedures approved by Massachusetts General Hospital’s Institutional Review Board (IRB #2014P001038). All procedures were in accordance with the Declaration of Helsinki. Scans were performed on a 3T Siemens Prisma scanner with a 64-channel head and neck coil. Anatomical scans used a 1 mm^3^ isotopic multi-echo MPRAGE [55]. Functional scans were acquired using single-shot gradient echo SMS-EPI^7^, with TR = 0.367 s, 2.5 mm^3^ isotropic voxels, 40 slices, Multiband factor = 8, matrix = 92 × 92, blipped CAIPI shift = FOV/4, flip angle = 32° to 37°, and no in-plane acceleration. EEG was acquired using MR-compatible 256-channel nets (Electrical Geodesics, Eugene, Oregon, United States of America) at a sampling rate of 1,000 Hz. EEG acquisition was synchronized to the scanner 10 MHz clock and the scanner cryopump was turned off during the scans to reduce the vibrational artifact. A reference layer made of vinyl and conductive satin was used to acquire reference signals for EEG noise removal [56].

#### Stimuli—Experiment 1

We presented a counterphase flickering radial checkerboard using Matlab and Psychtoolbox [57]. Each run lasted 254 s, with fixed 16 s ON and 16 s OFF periods. Participants were asked to fixate on a dot at the center of the checkerboard and press a button when they detected a color change. Several flickering frequencies were presented to subjects, ranging from 1 Hz to 20 Hz (1, 2, 4, 7.5, 12, and 20); we only analyzed stimulation frequencies of 4, 7.5, and 12 in these results, because insufficient trial numbers were obtained for the 1 and 2 Hz cases.

#### Data acquisition—Experiment 2

We analyzed a previously published dataset [34] of 20 healthy young adults (10 female; age range 19 to 36 years, mean = 25.15) who were selected because they had completed at least 1 run of a visual task with a fixed 16 s ON period. Twelve of the 20 subjects (6 female, age range 22 to 28 years, mean = 24.5) also had completed an additional version of the visual task with varying on-block durations ranging from 0.167 s up to 8 s. This subset of the subjects was included in the duration-dependence analysis. All subjects gave written informed consent to procedures approved by Massachusetts General Hospital’s Institutional Review Board (IRB #2014P001038). All procedures were in accordance with the Declaration of Helsinki. Scans were acquired on a 7T Siemens whole-body scanner with a custom-built 32-channel head coil. Anatomical scans were acquired with a 0.75 mm^3^ isotropic multi-echo MPRAGE [55]. Functional scans consisted of a single-shot gradient echo SMS-EPI at 1.1 mm^3^ isotropic resolution (TR = 1.11 s, TE = 26 ms, 38 slices, R = 4 acceleration, Multiband factor = 2, matrix = 174 × 174 full-Fourier, blipped CAIPI shift = FOV/2, flip angle = 70°).

#### Stimuli—Experiment 2

The same flickering checkerboard stimulus as in Experiment 1 was used. The ON block duration for the fixed duration condition was 16 s. The ON block durations for the variable duration condition were 0.167, 0.5, 1, 2, or 4 s. An 8 s duration condition was also present but was not collected for all subjects so it was excluded from the duration analysis.

#### Data acquisition—Experiment 3

We obtained written informed consent from 17 healthy young adults, and 16 were included in our final analysis (8 female, age range 19 to 31 years, mean = 23.8). One subject was excluded because they did not complete the full set of task runs. The protocol was approved by the Boston University Charles River Campus Institutional Review Board (IRB #5059E and #5023E). All procedures were in accordance with the Declaration of Helsinki. Participants were scanned on a 3T Siemens Prisma scanner with a 64-channel head and neck coil. Anatomical runs were acquired using a 1 mm^3^ isotropic T1-weighted multi-echo MPRAGE [55]. Functional runs were acquired using TR = 0.378 s, 2.5 mm^3^ isotropic voxels, 40 slices, Multiband factor = 8, blipped CAIPI shift = FOV/4, flip angle = 35° to 37°, and no in-plane acceleration. Additional sensors were used to record systemic physiology: Respiration was measured simultaneously using an MRI-safe pneumatic respiration transducer belt around the abdomen and pulse was measured with a photoplethysmogram (PPG) transducer (BIOPAC Systems, Goleta, California, USA). Physiological signals were acquired at 2,000 Hz using Acqknowledge software and were aligned with MRI data using triggers sent by the MRI scanner.

#### Stimuli—Experiment 3

We presented a counterphase flickering checkerboard visual stimuli using Matlab and Psychtoolbox [57]. The stimuli were presented on a VPixx Technologies PROPixx Lite Projector (VPixx Technologies, Quebec, Canada) with a refresh rate of 120 Hz. Subjects viewed the stimuli with a mirror that was mounted on the 64-channel head coil. Each run lasted 254 s, with fixed 16 s ON and 16 s OFF periods, beginning with an OFF period. To maintain attention, participants were asked to fixate on a dot at the center of the checkerboard and press a button on a response box when they detected a color change. The flicker frequency of the checkerboard varied (4, 8, 12, or 40 Hz) across runs.

### Data analysis

#### EEG preprocessing

EEG preprocessing was performed as described in detail in Fultz and colleagues [17]. Gradient artifacts were removed through average artifact subtraction [58] using a moving average of the previous 20 TRs. Electrodes were then re-referenced to the common average of EEG channels, excluding channels on the face and cheeks. A subset of the electrodes that were attached to an insulating reference layer (“reference electrodes”) rather than the scalp were re-referenced separately from the electrodes in contact with the head [56,59]. The signal from a subset of the reference electrodes was used in a dynamic sliding-window regression that was performed for each electrode individually using 30-s sliding windows to remove the ballistocardiogram artifact.

### EEG analysis

In Experiment 1, we selected the occipital EEG channel closest to Oz with good signal quality for each subject, and bandpass filtered the signal at the stimulus frequency for each run. We then extracted the instantaneous amplitude envelope of the band-passed signal using the Hilbert transform. For the cross-correlation analysis, the envelope of the EEG data was first downsampled to match the resolution of the upsampled fMRI data (upsampled sampling frequency = 4 Hz). Next, to generate a prediction of the BOLD signal, the amplitude envelope was convolved with a gamma function using the following equation:

$$h(t)=A*(\frac{t^{\alpha_{1}-1}\beta_{1}^{\alpha_{1}}e^{-\beta_{1}t}}{\Gamma (\alpha_{1})}),$$

where *α*_1_ = 6, *β*_1_ = 1, and Γ represents the gamma function [60]. *A* is the amplitude, which we set to equal 5. Finally, to produce a predicted CSF signal, we took the negative derivative of the estimated BOLD signal, approximating flow changes.

### MRI preprocessing

The cortical surface was reconstructed from the MPRAGE volume using recon-all from Freesurfer version 7 [61]. All functional runs were slice-time corrected using FSL version 6 (slicetimer; https://fsl.fmrib.ox.ac.uk/fsl/fslwiki) [62] and motion corrected to the middle frame using AFNI (3dvolreg; https://afni.nimh.nih.gov/). Each motion-corrected run was then registered to the anatomical data using boundary-based registration (bbregister) [63].

For the higher level FEAT analysis, functional runs were registered to 2 mm MNI common space and registrations were manually checked for accuracy. The linear functional to anatomical bbregister matrices were converted to FSL compatible matrices. The anatomical scan was then warped into 2 mm MNI space using nonlinear registration (FNIRT) that was initialized with linear transform matrices (FLIRT). Subject-level averages to the visual stimulus were extracted using a fixed effects model with the canonical double gamma hemodynamic response function and its temporal derivative. The group-level mean responses to the stimulus were extracted with a FLAME mixed effects model. Z-statistic images were thresholded using clusters determined by Z > 3.1 and a corrected significance threshold of *p* < 0.05.

### fMRI analysis

We defined the left and right hemisphere cortical gray matter regions of interest (ROIs) using the automatically generated Freesurfer-derived segmentations of cortical gray matter [61]. We also extracted the mean time series from the left and right primary visual cortex masks that were automatically generated by the cortical surface segmentation in Freesurfer [61]. Cortical time series were converted to percent signal change by dividing by the mean value of the time series after discarding the first 20 volumes to allow the signal to reach steady state.

### CSF flow signal extraction

To identify the CSF ROI, we manually traced the intersection of the fourth ventricle with the bottom slice of the imaging frame for each run for each subject. We extracted the mean signal in this region as the CSF inflow signal. All CSF inflow time series were converted to percent signal change by dividing by the value of the time series that represented the bottom 15th percentile value (since the CSF signal has a floor due to only measuring upwards flow). All time series were upsampled to a new sampling frequency of 4 Hz using spline interpolation before stimulus-locked averages were calculated.

#### Analysis of average evoked CSF flow (Experiments 1, 2, and 3)

We calculated the stimulus-locked CSF flow response to visual stimuli by averaging the CSF time series locked to each stimulus onset. We used a Wilcoxon signed-rank test to test whether the CSF inflow signal evoked by the neural manipulation was significantly different from baseline CSF inflow, by comparing the median CSF value in the 4 to 16 s range after stimulus onset, to the median CSF flow in the 4 to 16 s range after stimulus offset. To identify the specific time windows during which CSF flow increased significantly (S1 Fig), we combined the CSF data from all 3 experiments. We used a sliding window analysis to test whether each 1-s non-overlapping window was significantly different from the baseline flow, with the Wilcoxon signed-rank test. We used a Bonferroni corrected *p*-value threshold = 0.0024 (alpha level = 0.05, number of time windows = 21) to correct for multiple comparisons across time windows.

To test whether evoked CSF was more strongly driven by differences in visual cortex versus global cortex, we split all 1,001 trials from Experiment 3 into 2 categories (high-flow versus low-flow trials) using a threshold cutoff of 95% for high-flow trials. The threshold was set individually for each run as the 95% percentile of the time series. This threshold was chosen to give a roughly even split between the 2 categories (low trials *n* = 596, higher trials *n* = 405). The global cortical and visual cortical time series for each trial were averaged across the 2 categories.

### Analysis of CSF flow across stimulus durations (Experiment 2)

A subgroup (*n* = 12) of subjects in Experiment 2 viewed stimuli of multiple durations with long ISIs ranging from 17 s to 21 s, and these subjects were used in the duration analysis. To normalize the CSF signal across individuals, the bottom 15th percentile of values of a run was taken as the baseline. Each run time series was divided by the baseline to convert to percent signal change. Delta functions lasting 0.25 s each were placed beginning at stimulus onset and deconvolved to extract a trial response for each stimulus duration. We used the following windows around each stimulus onset to extract trial responses for cortical gray matter and CSF flow: [12 14 14 14 17] s for [0.17 0.5 1 2 4] s, respectively, to account for the variable trial lengths. Trial responses to each duration were averaged across all runs for all subjects.

To quantify differences in the magnitudes of CSF and BOLD trial response for each duration, we calculated the areas under the average response waveform separately for each duration. The area under the curve for CSF responses was calculated between t = 5 s and t = [12 14 14 14 17] s for the [0.17 0.5 1 2 4] s durations, respectively. The area under the curve for BOLD responses was calculated between t = 2 s and t = [12 14 14 14 17] s for the [0.17 0.5 1 2 4] s durations, respectively. CSF curves were baseline corrected using the value at t = 5 s, because this time corresponds to a period of maximally suppressed CSF flow. We calculated the difference in area between each stimulus duration (0.5, 1, 2, 4 s) and the shortest duration (0.17 s). To calculate 95% confidence intervals for the difference in the area under the curve, we generated 1,000 bootstrap samples, resampling over subjects, and repeating our area calculations on each sample. Confidence intervals were Bonferroni corrected for 4 comparisons.

To test whether evoked CSF flow and BOLD responses were significant, we tested whether the average deconvolved response function during a defined baseline period was significantly different from the average value during the response window for each subject using a paired *t* test. We defined “baseline” as t = 0 to t = 5 s for CSF traces, and t = 0 to t = 2 s for cortical BOLD traces. We averaged over the remaining time period to estimate the evoked response. For CSF, the response window was defined t = 2 s to t = [12 14 14 14 17] for the [0.17 0.5 1 2 4] s durations. For the cortical BOLD signal, the response window was defined t = 5 s to t = [12 14 14 14 17] for the [0.17 0.5 1 2 4] s durations.

### Analysis of CSF flow across stimulus frequencies (Experiment 3)

We calculated the group mean waveform for each stimulus frequency and smoothed the resulting waveform using a sliding 5-s window. BOLD and CSF waveforms were baseline corrected separately. The baseline value was taken at t = 2 s for the BOLD waveform and at t = 5 s for the CSF waveform. We calculated the area between the evoked CSF and BOLD curves in a 24-s window ([16 40] s for CSF, [2 26] s for BOLD). To create a confidence interval for the area, we drew 1,000 bootstrap samples for each frequency condition, resampling across subjects, and calculated the difference in area under the curve relative to the lowest frequency (4 Hz) for each bootstrap sample. We calculated the 95% confidence intervals using these bootstrapped samples, corrected for 3 comparisons (Bonferroni corrected).

### Systemic physiology analysis

We calculated the average CSF waveform locked to the cardiac cycle and breath cycle for each subject individually. We filtered the cardiac and breath traces between 0.6 to 1.3 Hz and 0.1 to 0.4 Hz, respectively, using zero-phase shift filtering. We extracted the phase and amplitude envelope of filtered cardiac and breath signals using the Hilbert transform and binned phases in bins of 20 degrees. We then calculated the mean CSF signal amplitude across each phase bin during task runs. To display relative timing (Fig 4), we estimated each subject’s average breath cycle and heart cycle in seconds by dividing the total number of cycles in each run by the total run time (e.g., 65 breath cycles/254 s = 4 s per breath cycle). To test for stimulus-locked changes in the amplitude of the respiratory belt or pulse-oximeter signals, we calculated the difference between the mean amplitude envelope of each signal during the stimulus on and off blocks in 1,000 bootstrapped samples and tested whether the 95% confidence intervals spanned zero.

## Supporting information

S1 Fig Measuring CSF flow in humans via MRI flow-related enhancement. (A) Tissue within the functional imaging volume (yellow) becomes saturated after experiencing multiple radiofrequency (RF) pulses. Fresh CSF (purple) that flows into the imaging volume has not experienced any RF pulses and can be detected as bright signals at the edge slices of the image volume, which is intentionally positioned in the fourth ventricle to capture fluid flow. The signal intensity changes from incoming fresh fluid are due to flow-related enhancement (FRE). (B) CSF (see arrow) is visible in the fourth ventricle as it flows upwards into the fourth ventricle. (C) Schematic of how CSF flow is reflected in fMRI FRE signals. Top—the true CSF velocity at the edge of the imaging volume consists of both positive (inflow) and negative (outflow) flow. Bottom—CSF measured via flow-related enhancement is only detected as it moves upwards into the imaging frame. Periods of outflow are not detected. Three example time points with no inflow (t1), slow inflow (t2), and fast inflow (t3) are indicated with dots. (D) Schematic of how flow signals appear in the edge slices of the imaging volume. Low-velocity flow (t2) is visible in only bottom slices as it travels slowly, experiencing an RF pulse and reaching steady state before the fluid reaches other slices. This results in bright flow signals in only the lowest slices of the volume. High-velocity flow (t3) travels across several slices before reaching steady state, as it flows farther before experiencing RF pulses. This results in bright CSF signals in several slices (t3; purple arrows).
(PDF)

S2 Fig Motion does not explain CSF inflow signals. (A) An example CSF trace across an entire run shows distinct inflow periods that do not overlap with periods of higher motion (framewise displacement > 0.1 mm; see arrows). (B) The distribution of correlation coefficients between CSF inflow traces and framewise displacement across all runs for all subjects show low correlations between flow and motion time series (mean correlation = 0.03).
(PDF)

S3 Fig Average CSF flow across all 3 experiments, zoomed into the stimulus off period (16–32 s). Average CSF flow across all subjects (*n* = 42), with no temporal smoothing. Shading indicates standard error across subjects. A sliding window analysis was used to test for significant flow changes in 1-s windows; green star indicates periods of CSF flow that differed significantly from baseline flow (*p* < 0.05, Wilcoxon sign-rank test).
(PDF)

S4 Fig High-flow trials versus low-flow trials show distinct dynamics in stimulus-evoked global BOLD signals. (A) Average CSF flow (purple) for high-flow trials (frames with flow above 95% percentile) and low-flow trials (dotted line). (B) Average global cortical BOLD responses sorted by high-flow and low-flow trials show a large difference in the cortical trace between trial types. (C) Average primary visual cortex BOLD responses sorted by CSF flow trial type show small differences between trial types. Error bars are standard error across trials.
(PDF)

S5 Fig Example EEG, V1, and CSF traces from 1 task run. Top: The bandpass-filtered EEG signal (blue) from an occipital channel in 1 example subject from the block design visual stimulus, flickering at 12 Hz. Overlayed on the filtered voltage trace is the amplitude envelope (orange) that shows the clear increases in the evoked EEG response during each stimulation period. Bottom: The CSF flow signal (purple) shows peaks following the decay of the cortical signal and is suppressed during on blocks when the cortical signal is high. The primary visual cortex (V1) signal (green) shows large responses to the visual stimulus as expected.
(PDF)

## Abbreviations

BOLD: blood-oxygenation-level-dependent
CSF: cerebrospinal fluid
EEG: electroencephalography
fMRI: functional magnetic resonance imaging
PPG: photoplethysmogram
ROI: region of interest
