# Correction: Neural activity induced by sensory stimulation can drive large-scale cerebrospinal fluid flow during wakefulness in humans

**DOI:** 10.1371/journal.pbio.3002123

**Published:** 2023-04-26

**Authors:** Stephanie D. Williams, Beverly Setzer, Nina E. Fultz, Zenia Valdiviezo, Nicole Tacugue, Zachary Diamandis, Laura D. Lewis

Author Laura D. Lewis’s affiliations are incomplete. The correct affiliations are as follows:

Athinoula A. Martinos Center for Biomedical Imaging, Massachusetts General Hospital, Boston, Massachusetts, United States of America

Department of Biomedical Engineering, Boston University, Boston, Massachusetts, United States of America
