# Supplementary material for: Neural activity induced by sensory stimulation can drive large-scale cerebrospinal fluid flow during wakefulness in humans
Source: PLoS Biol. 2023 Mar 30;21(3):e3002035. doi: 10.1371/journal.pbio.3002035 (PMC10062585; doi:10.1371/journal.pbio.3002035)
Supplement: S1 Fig — (A) Tissue within the functional imaging volume (yellow) becomes saturated after experiencing multiple radiofrequency (RF) pulses. Fresh CSF (purple) that flows into the imaging volume has not experienced any RF pulses and can be detected as bright signals at the edge slices of the image volume, which is intentionally positioned in the fourth ventricle to capture fluid flow. The signal intensity changes from incoming fresh fluid are due to flow-related enhancement (FRE). (B) CSF (see arrow) is visible in the fourth ventricle as it flows upwards into the fourth ventricle. (C) Schematic of how CSF flow is reflected in fMRI FRE signals. Top—the true CSF velocity at the edge of the imaging volume consists of both positive (inflow) and negative (outflow) flow. Bottom—CSF measured via flow-related enhancement is only detected as it moves upwards into the imaging frame. Periods of outflow are not detected. Three example time points with no inflow (t1), slow inflow (t2), and fast inflow (t3) are indicated with dots. (D) Schematic of how flow signals appear in the edge slices of the imaging volume. Low-velocity flow (t2) is visible in only bottom slices as it travels slowly, experiencing an RF pulse and reaching steady state before the fluid reaches other slices. This results in bright flow signals in only the lowest slices of the volume. High-velocity flow (t3) travels across several slices before reaching steady state, as it flows farther before experiencing RF pulses. This results in bright CSF signals in several slices (t3; purple arrows). (PDF) [file pbio.3002035.s001.pdf]

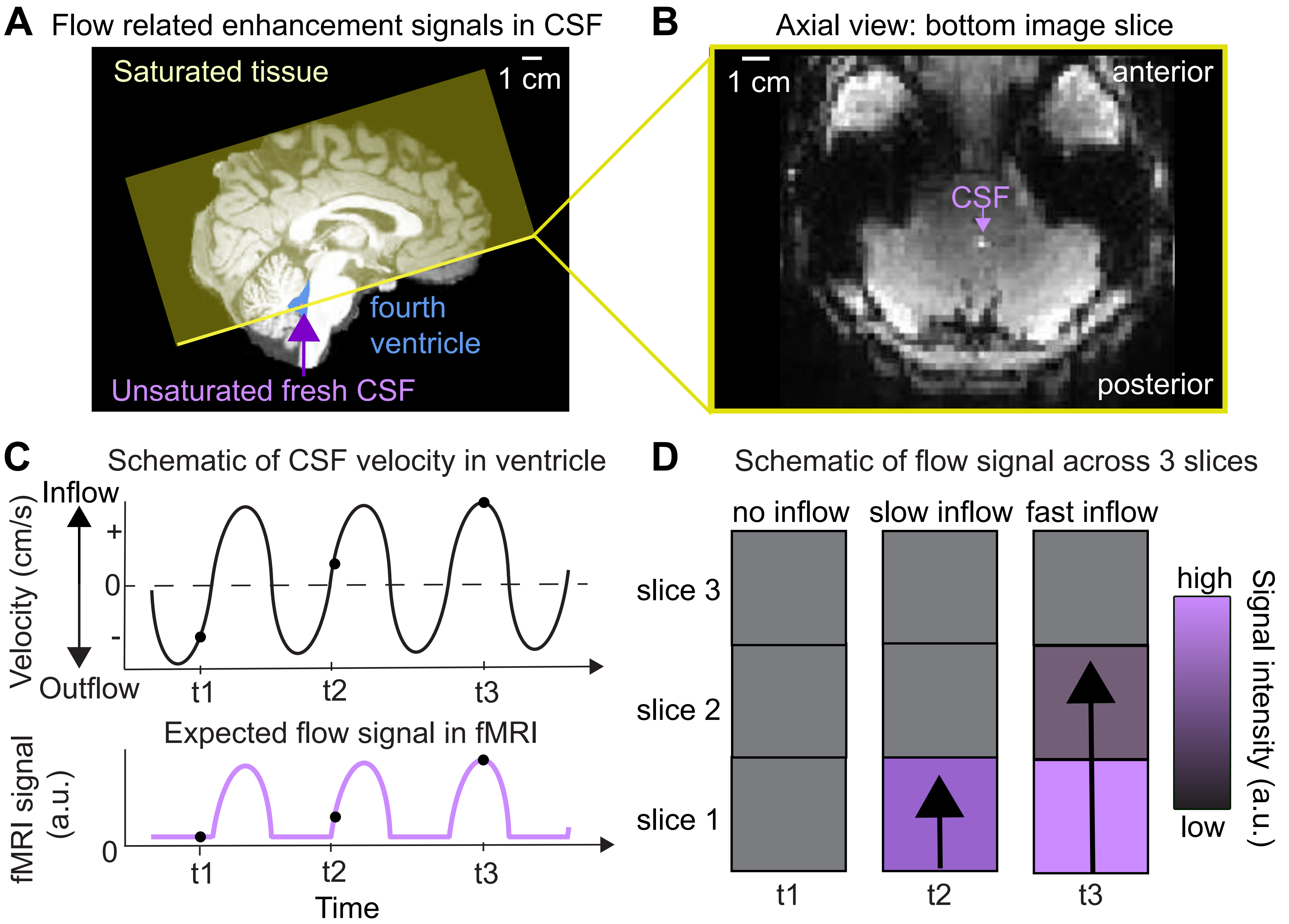
A
Flow related enhancement signals in CSF
Saturated tissue
1 cm
fourth
ventricle
Unsaturated fresh CSF
B
Axial view: bottom image slice
1 cm
anterior
CSF
posterior
C
Schematic of CSF velocity in ventricle
Inflow
Velocity (cm/s)
+
0
-
Outflow
t1
t2
t3
Expected flow signal in fMRI
fMRI signal
(a.u.)
0
t1
t2
t3
Time
D
Schematic of flow signal across 3 slices
no inflow
slow inflow
fast inflow
slice 3
slice 2
slice 1
t1
t2
t3
high
low
Signal intensity (a.u.)
