# Supplementary material for: Neural activity induced by sensory stimulation can drive large-scale cerebrospinal fluid flow during wakefulness in humans
Source: PLoS Biol. 2023 Mar 30;21(3):e3002035. doi: 10.1371/journal.pbio.3002035 (PMC10062585; doi:10.1371/journal.pbio.3002035)

A Example framewise displacement (FD) and CSF timeseries

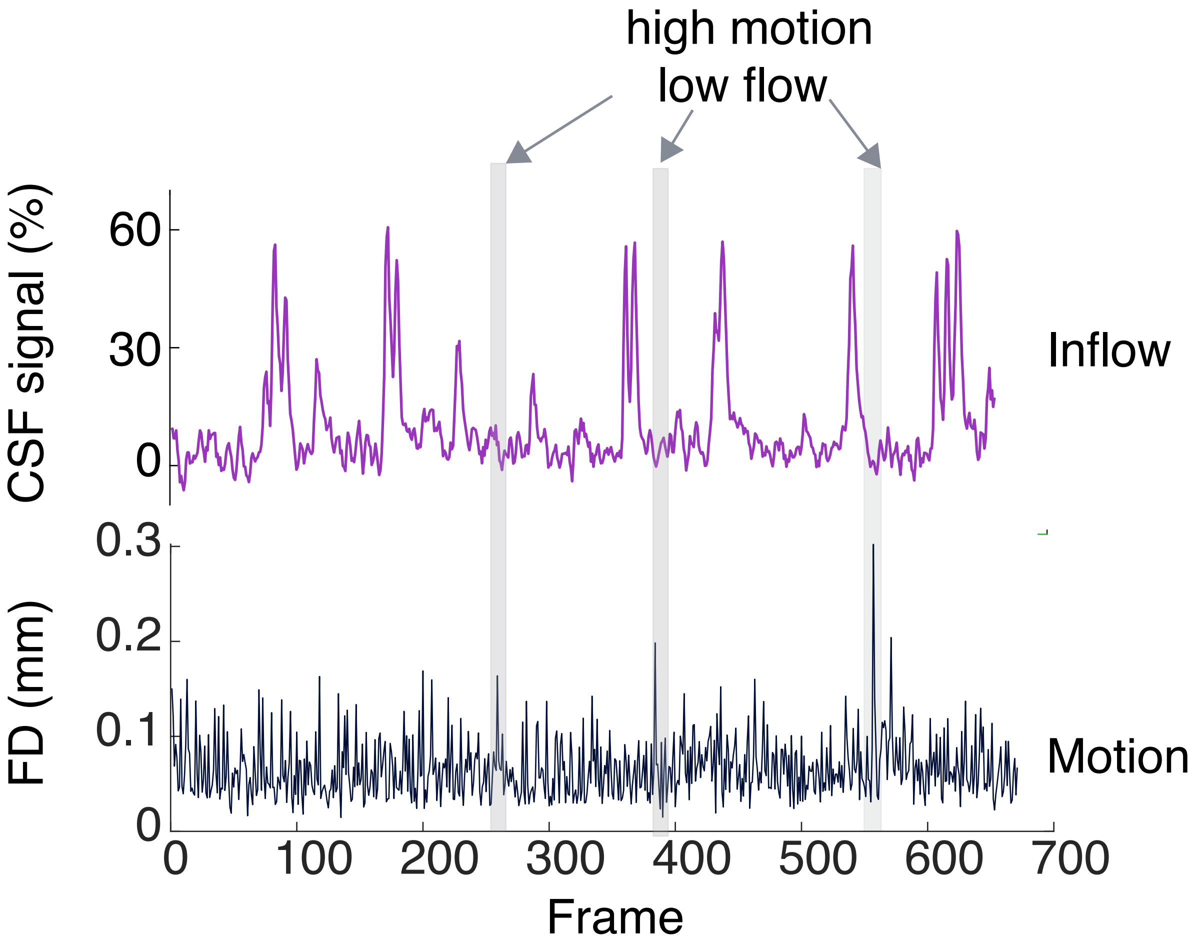

B Correlation between motion and CSF flow

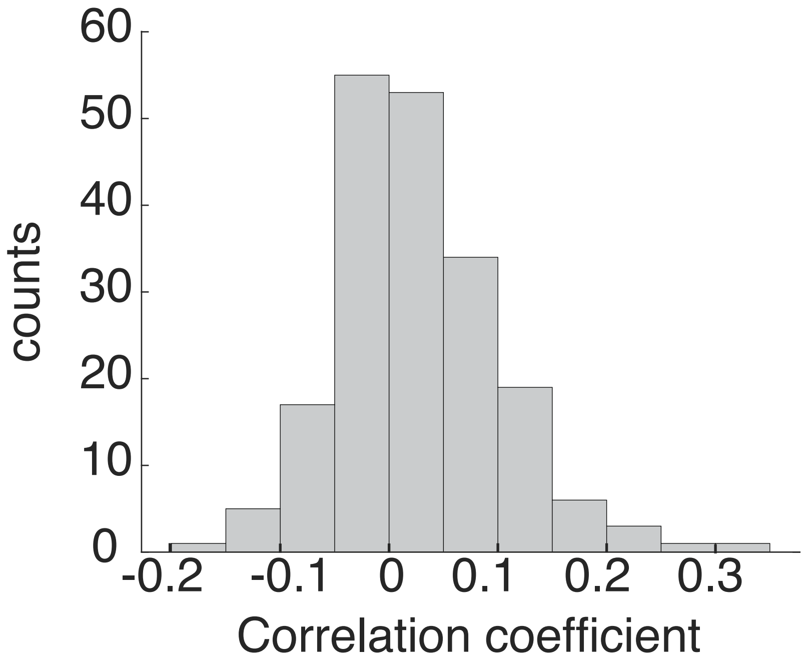

Supplement: S2 Fig — (A) An example CSF trace across an entire run shows distinct inflow periods that do not overlap with periods of higher motion (framewise displacement > 0.1 mm; see arrows). (B) The distribution of correlation coefficients between CSF inflow traces and framewise displacement across all runs for all subjects show low correlations between flow and motion time series (mean correlation = 0.03). (PDF) [file pbio.3002035.s002.pdf]
