# Supplementary figures and images for: Neural activity induced by sensory stimulation can drive large-scale cerebrospinal fluid flow during wakefulness in humans
Source: PLoS Biol. 2023 Mar 30;21(3):e3002035. doi: 10.1371/journal.pbio.3002035 (PMC10062585; doi:10.1371/journal.pbio.3002035)

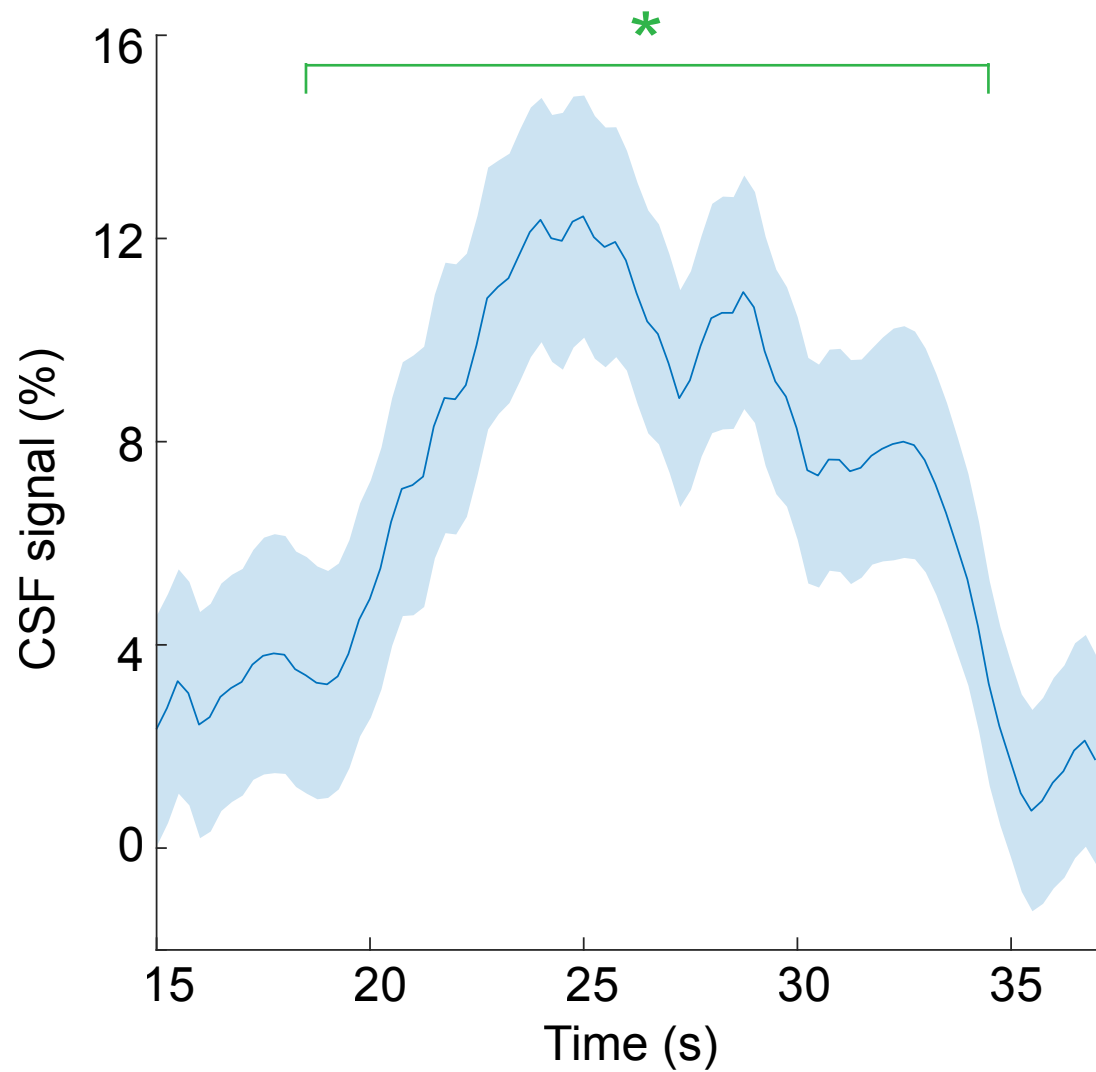

Supplement: S3 Fig — Average CSF flow across all subjects (n = 42), with no temporal smoothing. Shading indicates standard error across subjects. A sliding window analysis was used to test for significant flow changes in 1-s windows; green star indicates periods of CSF flow that differed significantly from baseline flow (p < 0.05, Wilcoxon sign-rank test). (PDF) [file pbio.3002035.s003.pdf]

**A**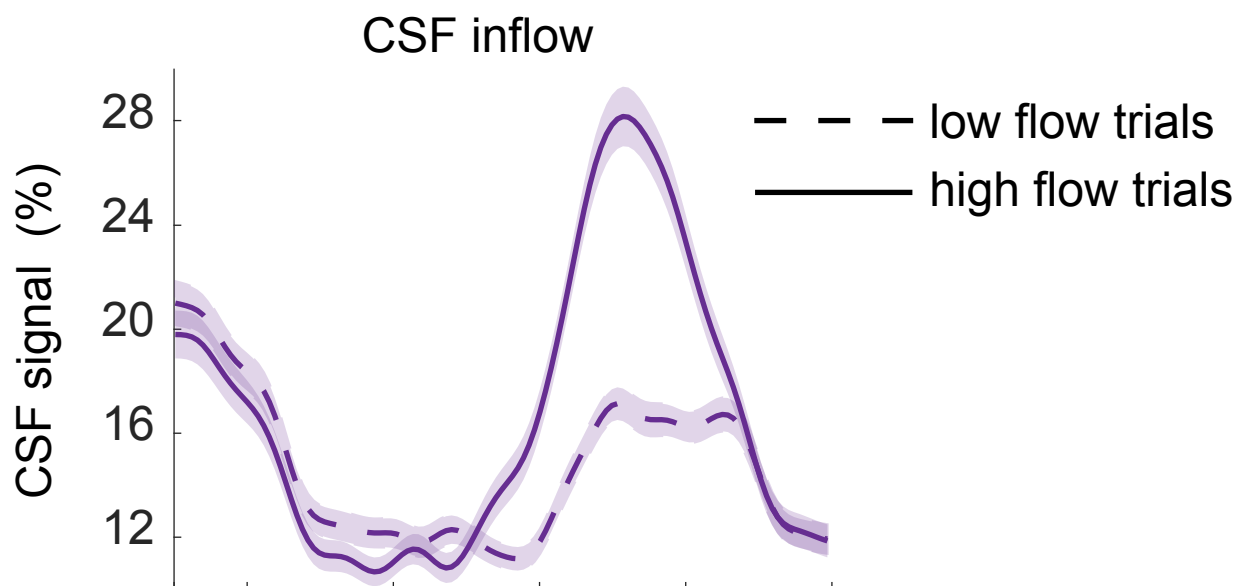**B**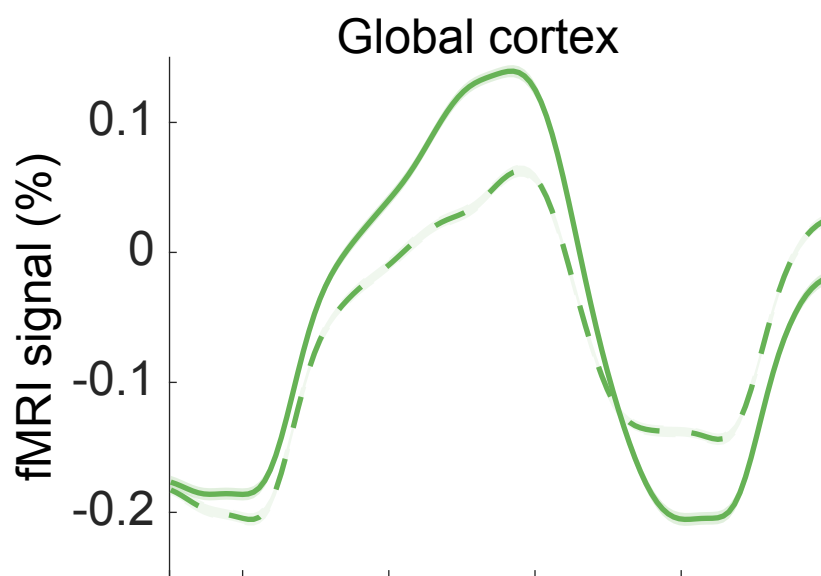**C**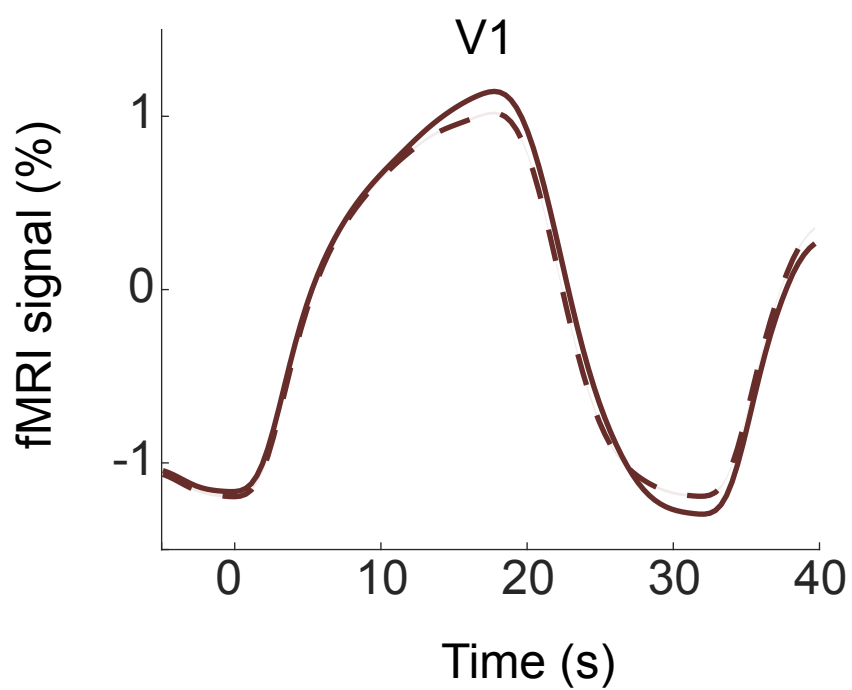

Supplement: S4 Fig — (A) Average CSF flow (purple) for high-flow trials (frames with flow above 95% percentile) and low-flow trials (dotted line). (B) Average global cortical BOLD responses sorted by high-flow and low-flow trials show a large difference in the cortical trace between trial types. (C) Average primary visual cortex BOLD responses sorted by CSF flow trial type show small differences between trial types. Error bars are standard error across trials. (PDF) [file pbio.3002035.s004.pdf]

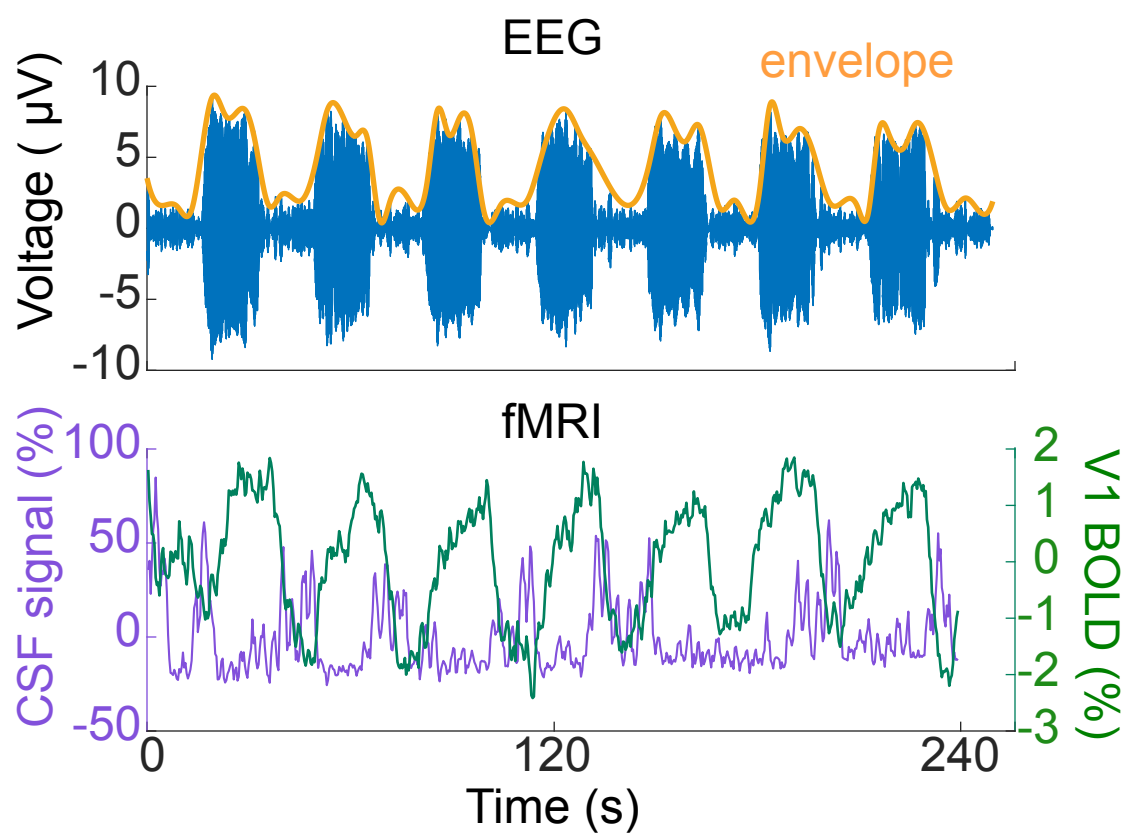

Supplement: S5 Fig — Top: The bandpass-filtered EEG signal (blue) from an occipital channel in 1 example subject from the block design visual stimulus, flickering at 12 Hz. Overlayed on the filtered voltage trace is the amplitude envelope (orange) that shows the clear increases in the evoked EEG response during each stimulation period. Bottom: The CSF flow signal (purple) shows peaks following the decay of the cortical signal and is suppressed during on blocks when the cortical signal is high. The primary visual cortex (V1) signal (green) shows large responses to the visual stimulus as expected. (PDF) [file pbio.3002035.s005.pdf]
